# A Thickness Illusion: Horizontal Is Perceived as Thicker than Vertical

**DOI:** 10.3390/vision3010001

**Published:** 2019-01-04

**Authors:** Jasper M. de Waard, Erik Van der Burg, Christian N. L. Olivers

**Affiliations:** 1 Bureau Roffa, 3023 DL Rotterdam, The Netherlands; 2 Department of Experimental and Applied Psychology, Vrije Universiteit, 1081 HV Amsterdam, The Netherlands; 3 Institute for Brain and Behaviour, 1081 BT Amsterdam, The Netherlands; 4 School of Psychology, University of Sydney, Sydney, NSW 2006, Australia

**Keywords:** illusion, vision, orientation, perception

## Abstract

We report two psychophysical experiments that investigate a visual illusion that is considered common knowledge among type designers, but has never been studied scientifically. Specifically, the thickness of a horizontal line is overestimated in relation to that of a vertical line. Experiment 1 confirmed the existence of the illusion. In Experiment 2, we replicated the effect and showed that the illusion is closely related to the vertical-horizontal illusion, in which the length of a vertical line is overestimated in comparison to a horizontal one. Both the overestimation of thickness and length is larger when the stimulus is surrounded by a horizontally elongated frame, as opposed to a vertically elongated frame. We discuss potential explanations for the thickness illusion and its relation to the vertical-horizontal illusion.

## 1. Introduction

**Figure 1:**
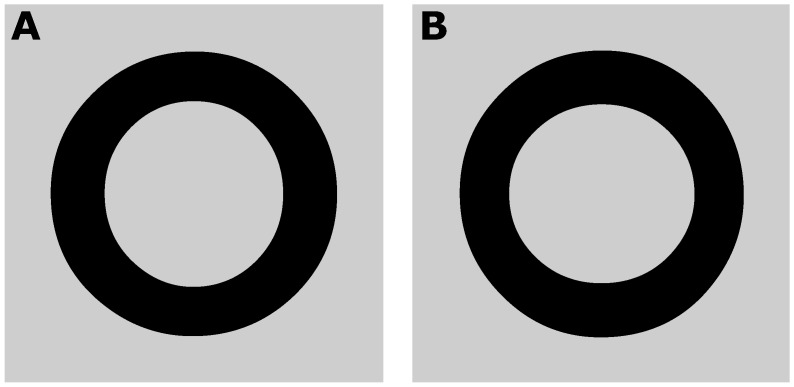
Irregular thickness of an ‘o’. (**A**) The ‘o’ shown upright. (**B**) The same ‘o’ rotated 90°.

Among type designers, it is common knowledge that even the most ordinary letters are subject to a multitude of illusions [1,2,3,4]. A skilled type designer is aware of these illusions, and knows beforehand to correct for them in the creation of a certain appearance. This study investigates one of the most common illusions apparent in type design, which affects the perceived thickness of lines. Specifically, the perceived thickness of a horizontal line is overestimated in comparison to a vertical line. For example, Figure 1A shows a lowercase ‘o’ that appears to have the same thickness all around. However, the difference in thickness becomes apparent in Figure 1B, where the same letter is rotated 90°. In order to appear to have a constant thickness, the vertically oriented segments of the ‘o’ have to be made thicker than the horizontally oriented ones, as is the case in Figure 1A. This particular illusion is demonstrated here in the form of a circle, but type designers compensate for the same illusion in rectilinear shapes, such as an ‘L’ or a ‘T’.

Although this thickness illusion is widely accepted in the type design community, to our knowledge it has never been scientifically demonstrated or investigated. The first of two experiments reported here therefore aimed to confirm its existence, and acquire an indication of its magnitude. In Experiment 1, we presented subjects on each trial with one horizontal and one vertical line of which we systematically varied the relative thickness, and asked them to judge whether the lines were equally thick or not. We expected the subjective point of equality to be biased towards thicker vertical lines.

Further motivation for choosing to investigate this illusion derives from the realization that it appears, a priori, to be similar in nature to a better-known illusion which *has* been studied before, namely the vertical-horizontal illusion [5] and in particular the anisotropy bias [6], which is about length rather than thickness. Specifically, this illusion concerns the overestimation of the length of a vertical line in relation to the length of a horizontal line. An overestimation of vertical length could relate to an overestimation of the thickness of a horizontal line in at least two ways. Firstly, both illusions could be the direct result of the same underlying anisotropy, in which visual input is being vertically stretched. Second, the thickness illusion could be the indirect consequence of the vertical-horizontal illusion: Because vertical lines are perceived as longer, they may be seen as relatively thinner (i.e., relative to their length). Künnapas [5] proposed that the vertical-horizontal illusion is the consequence of the horizontally elongated shape of the visual field. Künnapas obtained evidence for this theory, among other methods, by observing differences in the illusion magnitude when the stimulus was surrounded by vertically or horizontally elongated frames. Here, Experiment 2 investigated whether the same effects apply to the thickness illusion, and whether the effect sizes between the two illusions are correlated, which would suggest a shared cause. This experiment consisted of both a thickness and a length judgement task. Both tasks employed the same stimulus, in which the length and thickness of the lines varied independently of each other, to ensure that any observed differences were not caused by different stimuli. Moreover, within each task, we measured the effects of vertically versus horizontally elongated frames surrounding the stimuli. We hypothesized that the surrounding frame would affect the vertical-horizontal illusion and the thickness illusion in a similar fashion.

## 2. Experiment 1

**Figure 2:**
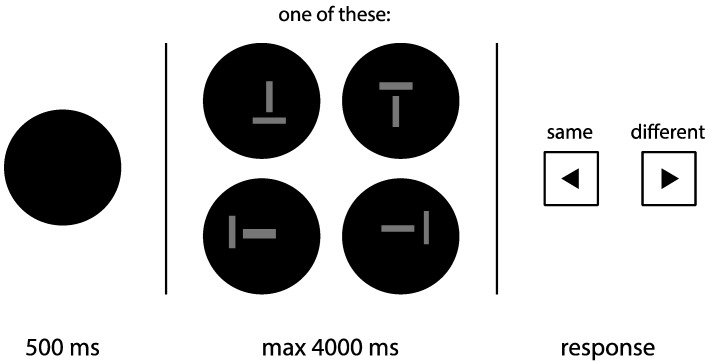
Schematic outline of a trial in Experiment 1. After a blank display (500 ms), a pair of lines (one vertical, one horizontal) were shown within a circular aperture, and subjects were instructed to judge whether the thickness of these lines was the same or not. The lines were shown until a response was provided (with a maximum duration of 4000 ms).

The aim of Experiment 1 was to provide scientific evidence for or against a thickness illusion—that is, a horizontally oriented line is perceived as being thicker than a vertically oriented line of the same thickness. On each trial, subjects viewed a horizontal and a vertical line, while the relative thickness of the lines was systematically manipulated. To exclude the possibility that thickness perception is mediated by length perception, the length of the horizontal line was varied randomly. Subjects were instructed to judge whether the thickness of the two lines was the same or not (see Figure 2 for an example trial). A similarity judgement task was used to avoid the possibility that subjects bias one line orientation over the other (i.e., a response bias; see references [7,8,9] for the same argument). Similarity judgement data then form a peaked distribution centered on the point of subjective equality (PSE), the point at which the two lines are perceived as equally thick. Specifically, we predicted the PSE to be significantly different from zero, in the direction of thicker vertical lines. 

### 2.1. Materials and Methods

#### 2.1.1. Subjects

Twenty-eight subjects (12 males; 16 females; ranging from 17 to 57 years; median age of 19) participated in Experiment 1. Given the novelty of the illusion, this relatively high number of subjects was planned beforehand. All subjects had normal or corrected to normal vision. Subjects with self-reported sensitivity to migraine or epilepsy were not allowed to participate. The research protocol complied with the ethical guidelines of the Faculty of Behavioural and Movement Science at the Vrije Universiteit Amsterdam, as assessed by the Scientific and Ethics Review Board (VCWE, file number VCWE-2016-215, approval date 2 January 2017).

#### 2.1.2. Apparatus

The experiment was programmed and run using Open Sesame 3.7 software [10]. The experiment was conducted in a dark room, and to avoid any influence from orientation cues, the monitor (Samsung 2233RZ; 1680 × 1050 pixels; 120 Hz refresh rate) was partially obscured by a black cardboard sheet with a circular opening of 16° (27 cm) in diameter, which was placed centered on the monitor. Subjects used a chinrest, so that the screen was perpendicular to the line of sight, at a distance of approximately 95 cm from the eyes.

#### 2.1.3. Stimuli

Each stimulus consisted of a grey (26 cd/m^2^) vertical and horizontal line on a black background (<0.5 cd/m^2^), placed in one of four different configurations (see Figure 2). The thickness of the lines varied from 0.6° to 1.1° (38 to 62 px). The length of the vertical line was 4.1° (240 px), while the length of the horizontal line was varied randomly between 3.7° and 4.5° (216 and 264 px, respectively). The distance between the two lines was varied randomly by 0.7° (40 px), to prevent subjects from attempting to assess the thickness of a line through its proximity to the other line. Furthermore, the location of the stimulus on the screen was varied randomly in a circular aperture of 1.7° (100 px) away from the center, to minimize retinal fatigue.

#### 2.1.4. Design and procedure

Subjects were verbally introduced to the experiment, followed by written instructions on the screen, and a practice round of 14 trials. Additional verbal instructions were provided when necessary. No mention was made of any illusion before or during the experiment. The experimental part consisted of 5 blocks of 104 trials. Per block, each configuration was used for 26 trials. On half of those 26 trials, the thickness of the vertical line was fixed at 0.9° (50 px), and on the other half, the thickness of the horizontal line was fixed at 0.9°. The varied line ranged in thickness from 0.5° to 1.2° (32 to 68 px), with steps of 0.05° (3 px). The order of trials was randomized within each block. Each level of difference in thickness occurred 8 times per block, so 40 times in total, evenly distributed over the different configurations. A trial started with the presentation of a black screen for 500 ms, after which the two lines were shown. Subjects were instructed to press the left-arrow or the right-arrow key if they perceived the lines as the same or not, respectively. The two lines were shown until subjects made a response, with a maximum of 4000 ms.

### 2.2. Results and Discussion

**Figure 3:**
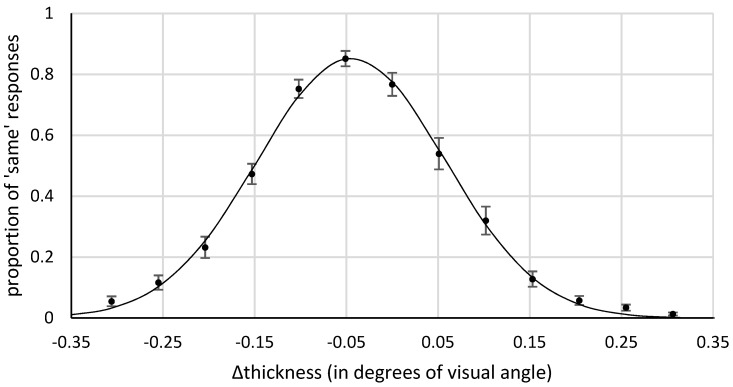
Results of Experiment 1. Mean proportion of ‘same’ responses as a function of difference in thickness (i.e., the thickness of the horizontal line minus the thickness of the vertical line). A negative value reflects a bias towards thicker vertical lines. The continuous line represents the optimally fitted Gaussian curve for the group mean data. The error bars reflect the standard error of the mean across subjects.

Practice trials and trials in which the time limit was exceeded (1.1%) were excluded from further analyses. Figure 3 shows the mean proportion of similarity responses as a function of the difference in thickness (the thickness of the horizontal minus the thickness of the vertical).

For each participant we fitted the response distribution with a Gaussian to estimate the point of subjective equality (PSE). The mean PSE was −0.05° (−2.71 px; SD = 0.03°, 1.52 px), with a mean bandwidth of 0.1° (6.03 px), and a mean amplitude of 0.85. This means that the vertical line must be 5.4% thicker than the horizontal line for them to be perceived as equally thick. This bias showed in 27 out of the 28 subjects. A one-sample, two-tailed *t*-test confirmed that the PSE differed significantly from 0, *t*(27) = −9.44, *p* < 0.001. A Bayesian *t*-test (one-sample, two-sided, Cauchy prior with center = 0, r = 0.707) yielded a BF of 4.6 × 10^6^ in favor of a bias.

Experiment 1 thus provides strong evidence for the existence of the thickness illusion. Horizontal lines are perceived as thicker than vertical lines—an illusion that has already been widely suspected within the type design community, and that proves substantial (at 5% distortion) and highly robust (with 27 out of 28 observers showing the bias).

One might argue that a line will appear thinner if it appears longer, so that extending a line would also have a thinning effect. If, in this way, the perception of line thickness is mediated by the perception of line length, it could be argued that the measured perceptual bias in Experiment 1 was an indirect effect of the vertical-horizontal illusion. In this scenario, vertical lines would be perceived as thinner *because* they are perceived as longer. To make sure that this was not the case, we randomly varied the length of the horizontal line, so that relative line length would be uninformative. As a further check, we used the following analysis. For each subject, we fitted a Gaussian curve using only those trials where the horizontal line was shorter than the vertical line (mean PSE = −0.04, SD = 0.03), and a Gaussian curve using only those trials where the horizontal line was longer (mean PSE = −0.05, SD = 0.02). A paired, two-tailed *t*-test confirmed that the PSEs did not differ significantly for these line lengths, *t*(27) = −1.310, *p* > 0.2; with a BF of 0.4 in favor of a difference. Note that this discarded possibility regards the modulated perception of thickness *through* length. As such, it is distinct from the possibility that estimation errors in both length and thickness perception are the result of the same underlying process in the visual system. Indeed, the question that remains is whether the thickness illusion and the vertical-horizontal illusion rely on the same underlying mechanism or not. Experiment 2 attempts to address this question.

## 3. Experiment 2

The aim of Experiment 2 was to examine whether the thickness illusion and the vertical-horizontal illusion are related. Such a relationship would be consistent with the same perceptual mechanism underlying the two illusions. 

Künnapas [5] believed that the vertical-horizontal illusion is, at least partially, caused by the shape of the visual field, which is wider than it is high. The edges of the visual field would provide a horizontally elongated visual context in which a vertical line takes up a bigger portion of the vertical space than an equally long horizontal line takes up of the horizontal space, thus leading to an overestimation of the length of the vertical line. Künnapas [5] obtained the first evidence for this theory by placing the stimulus into horizontally elongated, vertically elongated, and circular or square frames, which resulted in the illusion magnitudes varying accordingly. While Künnapas’ explanation of the vertical-horizontal illusion may not be the only one (an alternative will be treated in the General Discussion), the effect of the surrounding frame may still be employed to determine whether the vertical-horizontal and the thickness illusion are related. 

**Figure 4:**
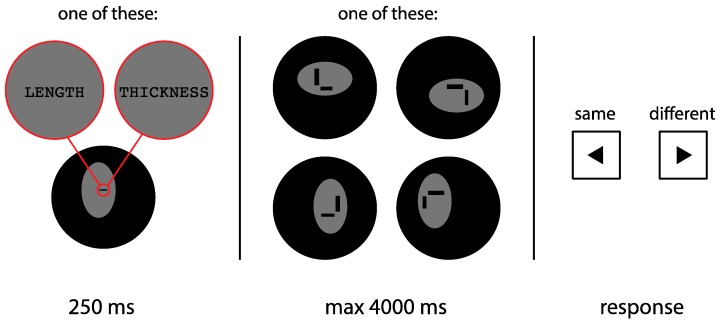
Schematic outline of a trial in Experiment 2. During a fixation period (250 ms), a horizontally or vertically oriented oval frame was already shown, along with the text ‘LENGTH’ or ‘THICKNESS’. Next, a pair of lines (one vertical, one horizontal) were shown within the frame. Depending on the task, subjects were instructed to judge whether the lines were equal in either thickness or length. The lines were shown until a response was provided (with a maximum duration of 4000 ms).

In Experiment 2, subjects were instructed to perform two separate tasks, revolving around either thickness or length. In the thickness task, subjects were instructed to judge whether the thickness of the horizontal and vertical lines was the same (as in Experiment 1). In the length task, subjects were instructed to judge whether the length of the two lines was identical or not. The two tasks were blocked and the order was counterbalanced across subjects. In addition, the stimuli in both tasks were placed in a horizontally or vertically elongated oval frame. The stimuli for both tasks were identical, so that any difference between the two tasks cannot be attributed to the stimuli used. An example trial is shown in Figure 4.

We expected to observe a thickness illusion when subjects perform a thickness task (replicating Experiment 1) and a vertical-horizontal illusion when subjects perform a length task (replicating, among others, [5,6,11,12,13]). More specifically, in accordance with Künnapas’ [5] findings, we expected the magnitude of the vertical-horizontal illusion to be larger when the stimuli were surrounded by a horizontally elongated frame than when they were surrounded by a vertically elongated frame. If the thickness illusion and the vertical-horizontal illusion rely on similar perceptual processes, then the magnitude of the thickness illusion should also be larger when the stimulus is surrounded by a horizontally elongated frame compared to a vertically elongated frame. In addition, if related, the magnitudes of the two illusions are expected to correlate across individual observers.

### 3.1. Materials and Methods

The method was the same as in Experiment 1, with the following exceptions. 

#### 3.1.1. Subjects

Thirty new students participated in the study (5 males; 25 females; ranging from 18 to 27 years; median age of 20). Given the uncertainty of the effect size of the frames on both the thickness and the vertical-horizontal illusion, this relatively high number of subjects was planned beforehand. All subjects had normal or corrected to normal vision, and subjects with self-reported sensitivity to migraine or epilepsy were not allowed to participate. The research protocol complied with the ethical guidelines of the Faculty of Behavioural and Movement Science at the Vrije Universiteit Amsterdam, as assessed by the Scientific and Ethics Review Board (VCWE, file number VCWE-2016-215, approval date 2 January 2017).

#### 3.1.2. Stimuli

Each stimulus consisted of a black vertical and horizontal line in one of four configurations, placed on a grey (26 cd/m^2^) horizontally or vertically elongated oval frame of 11.1° by 6.8° (650 by 400 px) on a black background (<0.5 cd/m^2^). Figure 4 illustrates a few example display configurations. In contrast to Experiment 1, here the line configurations were L-shaped, to rule out the possibility of a bisection bias having any effect on the perception of length [6]. The lines varied both in terms of length and thickness. In terms of thickness, either the vertical or the horizontal line was 0.9° (50 px) thick, with the remaining line between 0.6° and 1.1° (38 and 62 px) thick, in steps of 0.05° (3 px). This means that the difference in thickness between the two lines ranged from −0.2° to 0.2° (−12 to 12 px). In terms of length, either the vertical or the horizontal line was 3.3° (192 px) long, with the remaining line between 2.5° and 4.1° (144 and 240 px), in steps of 0.2° (12 px). This means that the difference in length between the two lines ranged from −0.8° to 0.8° (−48 to 48 px).

Similar to Experiment 1, the location of the two lines was slightly jittered in relation to each other by 0.1° (6 px), and the location of the stimulus and frame on the screen was varied randomly within a circular aperture of 1.4° (80 px) away from the center.

#### 3.1.3. Design and Procedure

During each task, subjects were either instructed to focus only on length, or only on thickness. Task type (length or thickness) was blocked, with two experimental blocks in total, of 576 trials each. Each experimental block was preceded by a practice round of 18 trials. The order of the tasks was counterbalanced across subjects. Per block, half of the trials (288) displayed a horizontally elongated frame, and the other half displayed a vertically elongated frame. Per frame condition, half of the trials (144) displayed a vertical line of 0.9° (50 px) thick, with the horizontal line varying in thickness, and the other half displayed a horizontal line of 0.9° (50 px) thick, with the vertical line varying in thickness. Furthermore, half of the trials (144) displayed a vertical line of 3.3° (192 px) long, with the horizontal line varying in length, and the other half displayed a horizontal line of 3.3° (192 px) long, with the vertical line varying in length. Variation in length was independent of variation in thickness. Each of the four line configurations (shown in Figure 4) occurred 144 times per block, evenly distributed over the horizontal and vertical frame conditions and the variation in length and thickness. The order of the trials was randomized.

Every trial started with a fixation period of 250 ms. During this period, the oval frame was already present, to maximize its impact on the perception of length and thickness. Furthermore, the text ‘LENGTH’ or ‘THICKNESS’ was displayed centrally on the screen, to remind subjects of the task. After the fixation period, the two lines were displayed. Subjects were instructed to press the left-arrow or right-arrow key if the two lines appeared to be the same or different (in terms of thickness or length, depending on the task), respectively.

### 3.2. Results and Discussion

Practice trials and trials in which the time limit was exceeded (0.2%) were excluded from further analyses. One participant had also participated in Experiment 1, so his/her data was discarded. The data of one other participant showed a fully inversed response pattern in the thickness task, indicating that he/she had systematically switched the response keys. For this participant, the responses were reversed before the analyses were conducted. 

**Figure 5:**
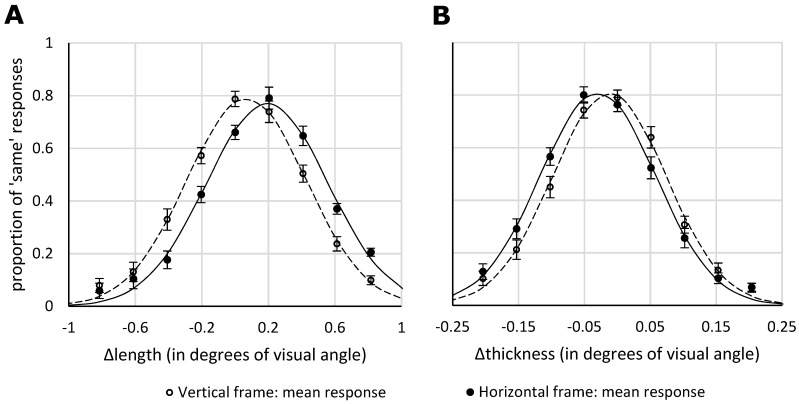
Results of Experiment 2. A negative value reflects that the vertical line was longer than the horizontal line, and vice versa. The continuous line represents the optimally fitted Gaussian curve for the group mean data. The error bars represent the standard error. (**A**) Mean proportion of ‘same’ responses as a function of difference in length (i.e., the length of the horizontal line—the length of the vertical line) for each frame orientation condition. (**B**) Mean proportion of ‘same’ responses as a function of difference in thickness (i.e., the thickness of the horizontal line—the thickness of the vertical line) for each frame orientation condition.

Figure 5 shows the mean proportion of ‘same’ responses as a function of difference in length (Figure 5A) and thickness (Figure 5B), for the two different frame orientations (horizontally or vertically elongated). 

For each subject, four Gaussian curves were fitted for each task (length or thickness) and frame condition (horizontal or vertical) separately.

#### 3.2.1. Length Task

As we sought to replicate the well-established vertical-horizontal illusion, and its sensitivity to frame orientation, we a priori chose one-sided statistical tests (but the results also stand under two-sided testing). Indeed, replicating the vertical-horizontal illusion, we found that the mean PSE in the length task was 0.1° (7.6 px), with a SD of 0.07° (4.4 px), and differed significantly from zero, as revealed by a one-sample one-sided *t*-test, *t*(28) = 9.25, *p* < 0.001. The length of the vertical line was overestimated compared to the horizontal line. A Bayesian version of the *t*-test (one-sample, one-sided, Cauchy prior with center = 0, r = 0.707) yielded a BF of 4.2 × 10^7^. Even in the vertical frame condition, where the illusion was weakest, the PSE differed significantly from 0 (*t* = 3.94, *p* < 0.001; BF = 62). Furthermore, and replicating reference [5], the vertical-horizontal illusion was larger when the stimuli were presented in a horizontal frame (PSE = 0.2°, 11.6 px) than when the stimuli were presented in a vertical frame (PSE = 0.1°, 3.6 px), which was reliable under a one-sided *t*-test, *t*(28) = 7.98, *p* < 0.001. A Bayesian version of this *t*-test (paired, one-sided, Cauchy prior with center = 0, r = 0.707) yielded a BF of 2.4 × 10^6^. The ratio of the illusion magnitude in the horizontal frame condition to that of the vertical frame condition was 1:3.3. The bandwidths for the horizontal (mean: 0.4°, 20.8 px) and vertical (mean: 0.4°, 20.6 px) frame condition did not differ significantly (*t* = 0.48, *p* > 0.6; BF = 0.2). The amplitudes for the horizontal (mean: 0.82) and vertical (mean: 0.84) frame conditions also did not differ significantly (*t* = 1.34, *p* > 0.1; BF = 0.4).

#### 3.2.2. Thickness Task

For the main effect we chose a one-sided test, as this concerned a replication of Experiment 1 (though the results also hold under a two-sided test). The mean PSE in the thickness task was −0.02° (−1.3 px), with a SD of 0.02° (1.1 px), and significantly different from zero, as revealed by a one-sample one-sided *t*-test, *t*(28) = −6.62, *p* < 0.001. Replicating Experiment 1, we found that the horizontal line was perceived as thicker than the vertical line. The Bayesian version of the *t*-test (one-sample, one-sided, Cauchy prior with center = 0, r = 0.707) yielded a BF of 93 × 10^3^. In the vertical frame condition, where the illusion was weakest, the PSE still differed significantly from 0 (*t* = −3.9, *p* < 0.001; BF = 56). Furthermore, the thickness illusion was larger when the stimuli were presented in a horizontal frame (PSE = −0.03°, or −1.8 px) than when the stimuli were presented in a vertical frame (PSE = −0.01°, or −0.1 px), as revealed by a paired-samples two-sided *t*-test, *t*(28) = −9.75, *p* < 0.001. A Bayesian version of this *t*-test (paired, two-sided, Cauchy prior with center = 0, r = 0.707) yielded a BF of 6.1 × 10^7^. Here, given the novelty of the finding, two-sided testing was chosen. The ratio of the illusion magnitude in the horizontal frame condition to that of the vertical frame condition was 1:2.3. The bandwidths for the horizontal (mean: 0.1°, 5.1 px) and vertical (mean: 0.1°, 4.9 px) frame condition did not differ significantly (*t* = 1.29, *p* > 0.2; BF = 0.4). The amplitudes for the horizontal (mean: 0.85) and vertical (mean: 0.84) frame condition also did not differ significantly (*t* = 0.404, *p* > 0.6; BF = 0.2).

#### 3.2.3. Correlations

**Figure 6:**
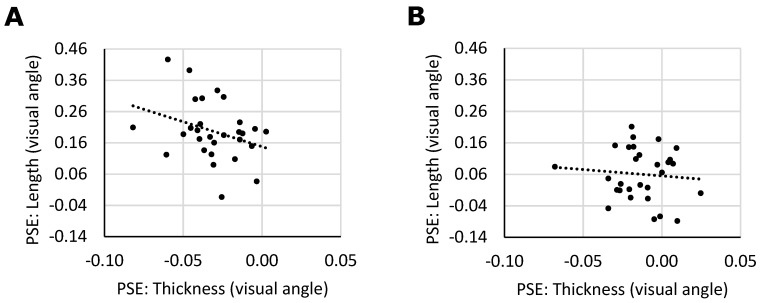
Correlation between the thickness illusion and the vertical-horizontal illusion. (**A**) Correlation between the PSE in the length task versus the PSE in the thickness task in horizontal frame condition. (**B**) Correlation between the PSE in the length task versus the PSE in the thickness task in vertical frame condition.

We were interested whether individuals showing a larger vertical-horizontal illusion would also show a larger thickness illusion. The signs of the vertical-horizontal and the thickness illusion were defined as positive and negative values, respectively. As a result, a negative correlation is expected if both illusions share underlying processes. Given that both the thickness illusion and the vertical-horizontal illusion were predominantly present when the stimuli were presented within the horizontally elongated frame, we computed the correlations for each frame type separately (Figure 6). We indeed observed a significant correlation between the length and the thickness illusion (*p* = 0.045, r = −0.321, BF = 1.73) when the frame was horizontal, but no such correlation when the frame was vertical (*p* = 0.323, r = −0.089, BF = 0.34) as would be expected given that here the illusions were weak in the first place.

Thus, in Experiment 2, the vertical-horizontal illusion as well as the thickness illusion were evident in the data. Furthermore, both illusions were affected by the visual reference frame, as they were both significantly reduced when presented within a vertically elongated context. The similarity of both illusions is further reflected in two other findings. Firstly, the two illusions show a comparable order of magnitude. The effect size was 3.9% in the vertical-horizontal illusion, and 2.6% in the thickness illusion. The ratio of the illusion magnitude in the horizontal frame condition to that of the vertical frame condition was 1:3.3 in the vertical-horizontal illusion and 1:2.3 in the thickness illusion. While these differences are not negligible, they do not suggest a fundamental difference either. Secondly, within the condition in which the illusion was the strongest (the horizontal frame condition), we found that individual variation in the PSEs of the two illusions correlated significantly. When taken together, these findings support the hypothesis that the two illusions rely on similar perceptual mechanisms.

## 4. Discussion

The two experiments in this study have revealed several findings. First and foremost, horizontal lines are perceived as thicker than vertical ones, an effect we have called the thickness illusion. This illusion, while a normal part of type designers’ everyday practice, has until now, to our knowledge, never been experimentally established. Experiment 1 provided convincing evidence for the illusion, which was replicated in Experiment 2. Second, we argue that the cause of the thickness illusion is likely to be related to that of the vertical-horizontal illusion (in which vertical length is overestimated relative to horizontal length). Experiment 2 showed that both illusions were found to be larger when they were shown in a horizontally elongated frame, as opposed to a vertically elongated frame. The similarity between the two illusions is further suggested by the similar effect sizes, and the correlation across individual variation (for horizontal frames, when the illusions were strongest).

The question remains what the cause of the vertical-horizontal illusion, and by extension likely also that of the thickness illusion, may be. As discussed earlier, Künnapas [5] has proposed that the vertical-horizontal illusion is caused by the anisotropy of the visual field, specifically its horizontal elongation (see reference [14] for a similar account in which the anisotropy is in the visual orientation filters). Interestingly, he found that adjusting the visual field so that it becomes vertically elongated strongly decreases the illusion, but does not entirely reverse it [12]. While this may reflect a difference between short and long-term effects of the visual field or reference frame, a different explanation has recently garnered support. Howe and Purves [15] argue that the illusion is the consequence of the visual system’s way of dealing with the inverse problem (which holds that it is impossible to deduce the properties of the three-dimensional reality from a two-dimensional projection on the retina). In their view, the visual system makes a probabilistic analysis of the length of lines in three-dimensional space, based on the two-dimensional projection on the retina and previous experience with lines of various orientations in natural scenes. Interestingly, they established that the overestimation of length in the vertical-horizontal illusion is actually not the largest for vertical lines, but for lines that deviate 20 to 30 degrees from vertical. They also took photographic images of outdoor scenes in combination with a laser-based range finder that enabled them to determine the real-world distance behind each pixel value. This way they could compute two-dimensional as well as three-dimensional lengths for lines present in the images. They found that the normalized mean ratio of three-dimensional to two-dimensional length as a function of line orientation bore a striking similarity to the mean *perceived* line length as a function of orientation (see Figures 1 and 3C in reference [15]). That is, the ratio of the real three-dimensional line length to the two-dimensional image projection was on average largest for 20 to 30 degrees from vertical, leading Howe and Purves [15] to conclude that the length illusion reflects these probabilistic properties of the visual world.

Given the similarities between the vertical-horizontal illusion and the thickness illusion it could well be that Howe and Purves’ theory also generalizes to the thickness illusion reported here. This would make sense theoretically, since both thickness and length perception suffer from the same inverse problem, which means the visual system could apply the same solution to both cases. An implication would be that the thickness illusion, rather than being merely about vertical and horizontal lines, follows a different orientation distribution. However, this hypothesis remains to be tested. Evidence to support the idea that both length and thickness perception rely on probabilistic properties of the natural environment may be obtained firstly by investigating the perception of line thickness as a function of more orientations than just vertical and horizontal. If the vertical-horizontal illusion and the thickness illusion are indeed closely related, the resulting function could appear much like the inverse of Figures 1 and 3C in reference [15]. To come to a more definitive conclusion, the probabilistic relation between the two-dimensional and three-dimensional thickness of lines in natural scenes could be investigated, though a computational operationalization of line thickness may prove to be more difficult than of length.

Besides the exact cause of the thickness illusion, some other questions also remain to be answered. Firstly, does the illusion persist in natural scenes, in stimuli other than abstract lines? Furthermore, it would be interesting to know whether the context of typography, as opposed to non-typographical lines, influences the magnitude of the illusion. The present study consciously avoided any context of typography, but this is not by any means a necessity. Of course, typefaces exist in many different styles, the majority of which do not try to achieve a constant visual thickness—whether physically or perceptually. Nevertheless, geometric typefaces generally do avoid visual differences in thickness, thus forming a relevant testcase. Interestingly, the effect size in the present study (5% in Experiment 1 and 3% in Experiment 2) is much smaller than the thickness difference between vertical and horizontal lines in some of the best-known geometric typefaces, such as Futura (13%) and Avenir (20%). This discrepancy may be explained by an additional influence of typographic context on thickness perception, but it could also be that factors such as size and acuity influence the magnitude of the illusion.

Taking the context of typography one step further, it could also be possible that the illusion can be influenced by the use of different kinds of writing systems. An interesting finding from the field of type design is that while Latin typefaces commonly have thicker vertical than horizontal lines, Arabic typefaces are commonly thicker in horizontally oriented segments, even when the intended result is to appear constant in thickness [16]. This would imply a cultural influence on the perception of thickness, which itself may or may not be based on the properties of the writing system.

## 5. Conclusions

The two experiments in this study have served to show several things. Firstly, horizontal lines are perceived as thicker than vertical ones, an effect here called the thickness illusion. Convincing evidence for this illusion was initially obtained in Experiment 1, and replicated in Experiment 2. Experiment 2 compared the thickness illusion to the vertical-horizontal illusion, in which the length of a vertical line is overestimated in comparison to a horizontal one. Both illusions were found to be larger when they were placed in a horizontally elongated frame, as opposed to a vertically elongated frame. The similarity between the two illusions is further suggested by the findings that the effect sizes of both illusions are on a comparable order of scale and that the illusion magnitudes per individual are significantly correlated with each other within the horizontal frame condition.

We believe the most probable cause for the vertical-horizontal illusion, and by extension also the thickness illusion, lies in a probabilistic estimation of three-dimensional length, based on the visual properties of natural surroundings. Because it is confronted with the inverse problem (it is impossible to deduce three-dimensional properties from a two-dimensional retinal projection), the visual system interprets lines of varying orientations according to a likelihood distribution based on previous experience. In turn, this distribution is derived from the ratio of two-dimensional to three-dimensional length for lines in the natural visual world, which may include writing systems.
